# Detecting unacceptable behavior of an autonomous vehicle using electroencephalography

**DOI:** 10.1038/s41598-025-18305-2

**Published:** 2025-09-12

**Authors:** Maren A. K. Bertheau, Christoph S. Herrmann

**Affiliations:** 1 Department of Psychology, Experimental Psychology Lab, Carl-von-Ossietzky University, Ammerländer Heerstr. 114-118, 26111 Oldenburg, Germany; 2 Department of Informatics, Karlsruhe Institute of Technology (KIT), Am Fasanengarten 5, 76131 Karlsruhe, Germany; 3 European Medical School, Cluster for Excellence “Hearing for All”, Research Center Neurosensory Science, Carl-von-Ossietzky University, Carl-von-Ossietzky-Straße 9-11, 26129 Oldenburg, Germany; 4 Research Center Neurosensory Science, Carl von Ossietzky University, 26129 Oldenburg, Germany

**Keywords:** Autonomous driving, ERP, N1, N2, P2, P3, Psychology, Human behaviour, Neuroscience, Neuronal physiology, Social neuroscience

## Abstract

In the present study, we investigated event related potentials (ERPs) in the context of autonomous driving - specifically in left-turn situations through oncoming traffic. We recorded electroencephalography while participants (n = 33) observed a simulated autonomous vehicle executing a left turn maneuver through oncoming traffic. In the ERP, we observed an increased N2 (251 to 431 ms) when the AV behaved incongruently to the participant’s assessment of the turn situation compared to when it behaved congruently. There were no significant effects in N1 (142 to 182 ms), P2 (177 to 237 ms), nor P3 (439 to 689 ms). This suggests that, in human-AV interaction interaction, ERP-based devices might, in the future, be able to identify critical situations. However, further research is needed to bring current findings from fundamental research closer to application.

## Introduction

Highly autonomous vehicles (AVs) (SAE level 4) are becoming increasingly prevalent. Highly and fully AVs (SAE level 4 & 5) bear a great potential for increased road safety, energy efficiency, and social participation for people without driver’s licenses, for instance, underage, elderly, or individuals with disabilities^1^. However, even when federally approved for real-world traffic, an AV’s non-human nature may lead to behavior that is formally lawful, but not necessarily socially or humanly acceptable. For instance, unacceptable behavior by the AV ccould arise in novel circumstances that the AV has not encountered before^2^ or when its behavior would not fully align with the users’ individual attitudes, personality, risk aversion, driving experience, etc.^3^. Additionally, AVs can take information from sensors beyond human sensory abilities into account (e.g., radar, infrared, etc.)^4,5^. With these advantages, they would be able to perform certain traffic maneuvers more safely and swiftly than a human driver^4,6^. Consequently, the behavior of an AV, even with federal approval, could at times be unexpected or unpredictable and thus likely unacceptable to the human user.

In line with the ’Autonomous Vehicle Acceptance Model’^7^ such unexpected or unacceptable behavior could induce stress and anxiety^8^ and lead to distrust in AVs, and thus to a refrain from using AVs^7^. A system could attempt to remedy this by adequately and individually adjusting its behavior by, e.g., giving an explanation^9^. However, for these adjustments to be successful, timing is crucial^10^. Therefore, critical or unacceptable situations need to be identified, especially in time-critical maneuvers.

To translate this complex need into simplified experimental terms, we define unacceptable situations as those in which an AVs exhibits behavior incongruent with the user’s expectations. From an experimental design perspective, this is advantageous because it allows the creation of equal numbers of trials between experimental (incongruent) and control (congruent) conditions, independent of individual preferences.

In time-critical driving maneuvers, it is impractical to measure user perceptions (or ratings of acceptability) of AV behavior via questionnaires^11^. Physiological measures may provide an unobtrusive way to assess user perceptions of AV behavior during dynamic and complex driving situations^11^. In particular, electroencephalography (EEG) has cost-efficient mobile solutions, e.g.,^12^, and a high temporal resolution that is well suited to investigate human - AV interaction in time-critical traffic situations. A frequently used measure in the EEG is the event related potential (ERP),a brain response time-locked to an event such as the presentation of a stimulus. These signals occur within (ms) and precede any voluntary motor reaction^13^.

When reviewing previous studies on the acceptability of decisions by AVs in traffic situations, to the best of our knowledge, EEG has never been thoroughly utilized before. Yet, there are several ERPs components with systematic amplitude modulations in tasks requiring similar cognitive processing as those required for assessing the incongruence of an AV’s behavior. In foundational ERP research, there is a wide variety of frequently used tasks to manipulate congruence and incongruence. In the visual domain, commonly used tasks are the Eriksen Flanker task^14^, the Stroop task^15^, as well as Decision Making or Estimation tasks, e.g.,^16–19^. Incongruent Eriksen Flanker (e.g., HHSHH click S) as well as incongruent Stroop trials (e.g., “red” printed in blue) evoke an increased fronto-central N2^20^ and modulate the parietal P3 response in the ERP^20,21^. These ERPs might reflect the increased cognitive control^20^, specifically inhibitory control or response inhibition^20,22^. In loss trials from Decision Making or Estimation Tasks,^16–19^ (where the outcome was incongruent to a participant’s expectation), the ERP shows a feedback related negativity (FRN), a fronto-central negative deflection peaking around 200–300 ms after negative feedback^23–26^, which is then followed by a frontal P3a at around 300-400 ms and a parietal P3b at around 400-500 ms^26^. The FRN is thought to play a central role in reinforcement learning processes^26–28^ and is functionally interpreted as tracking the difference between actual and expected outcomes, referred to as reward prediction errors^28^

Admittedly, all of these tasks are extremely simplified in comparison to traffic situations. Nevertheless, the underlying mechanisms of cognitive control, such as conflict resolution and retrieval, are highly relevant to successfully assess complex traffic maneuvers such as turning left through oncoming traffic. When choosing whether to turn left through a specific gap, conflict resolution is highly relevant. Every new gap needs to be assessed so that users can expect how the specific gap will be handled by the AV. Subsequently, users would need to retrieve their expectations to assess whether it was congruent or incongruent to the AV’s behavior.

Folstein & van Petten^20^ summarized that both N2 and P3 are broadly speaking associated with the significance of events (e.g., relevant stimuli). The N2 is generated in three distinct brain regions that are believed to serve different cognitive functions. The anterior N2 is associated with novelty and mismatch in attended stimuli. Further, the fronto-central is N2 associated with response inhibition (Go-No Go task) and the posterior N2 is associated with visual attention^20^. However, while functionally distinct from the view of basic research, all these paradigms and mechanisms have in common that the N2 marks significant stimuli that are either new, rare, attended/awaited, or errors. The P3 is commonly separated into a more frontally generated P3a and a more parietally generated P3b. The P3a is usually associated with orienting attention to unexpected or significant events, whereas the P3b is associated with updating of working memory^20^. Since we are interested in the unexpected behavior of AVs, and based on our previous research^29^, we would expect an increased P3a in response to incongruent trials. Hence, we suspect that the ERP ccomponents N2 and P3 might play a role when an AV behaves incongruently to users’ expectations in traffic. Further, earlier components could also be involved. The N1 is thought to play a crucial role in visual attention and serves as an index of discrimination processes^30^. The prefrontal P2 is thought to play a role in stimulus-response mapping, in other words, classifying stimuli and matching them to the correct response^31^.

For the present study, we aimed to investigate the feasibility of using the ERP technique to capture participants’ acceptance of AV behavior in dynamic traffic situations simulated in a laboratory environment. Our results might open new avenues to unobtrusively evaluate reciprocal communication between human users and AVs. Thus, this work is at the intersection of basic psychophysiological research and applied human-machine interaction development.

Hence, we developed a left turn task using driving simulations in a laboratory environment to effectively investigate ERPs during autonomous driving maneuvers. Driving simulations are advantageous as they provide a high control over the whole experimental situation as compared to e.g., Wizard of Oz approaches, where participants believe to interact with an actual AV in the field, but which is actually (sometimes remotely) operated by an unseen human confederate: see e.g.,^32^. Further, the electrically shielded laboratory environment provides a higher EEG data quality as compared to field setups^33^.

We investigated left turn situations through oncoming traffic because they are (1) a frequently occurring traffic maneuver, (2) a high risk for accidents, (3) a known difficulty for AVs, and (4) allow for personal opinions or style. (1) The frequency of this maneuver is relevant, because for reliable ERP results it is necessary to plausibly repeat a certain task/situation numerous times^33^. (2) To exemplarily showcase the proneness for accidents of left turn maneuvers, note that in 2023 there were 97.481 turning, turning-in, and crossing accidents with personal injury on German roads, which accounts for 34% of total accidents that year^34^. (3) Furthermore, Wiegand, Eiband, Haubelt & Hussmann^10^ identified turning situations to cause existing AVs to behave unexpectedly using thematic analysis of real-world experience reports. (4) What makes turning left especially interesting to us, is that even if the maneuver is carried out without an accident, there is still room for personal preferences: Some vehicle users are more risk averse than others and therefore deem different gap sizes in the oncoming traffic as save enough, hence their assessment might frequently differ from the AV’s.

In our left turn task, participants indicated whether they would take a left turn when there were gaps of variable length in oncoming traffic in a left turn situation. Next, the participants observe the AV’s behavior as it either executes the turn or waits. This behavior can align (“congruent”) or conflict (“incongruent”) with the participant’s decision.

We hypothesized that participants would rate congruent trials as more acceptable than incongruent ones (*Acceptability*).

Regarding the physiological measure, EEG, we hypothesized that the amplitudes of the N2, and P3 components of the ERP would be greater after the presentation of the AV’s behavior in incongruent trials (see “Methods”) as compared to congruent trials. Additionally, since we use dynamic rather than static stimulus material, we wanted to ensure that we were replicating the temporal sequence of ERP components that are typically observed in response to viewing static images. Hence, we also included earlier components in our analysis and expected increased N1 and P2 amplitudes in incongruent trials compared to congruent trials.

## Results

### Behavioral results

After artifact exclusion, $98.70 \% (\bar{x} =197.39(standarddeviation(SD)=2.62)$ per participant) trials went into analysis. In $69.95 \% (\bar{x} = 137.91 (SD=46.90))$ of trials, participants indicated that they wanted to turn and vice versa in $30.05 \% (\bar{x} = 59.55 (SD=47.75))$ that they wanted to wait (see supplementary Table 1).

**Fig. 1:**
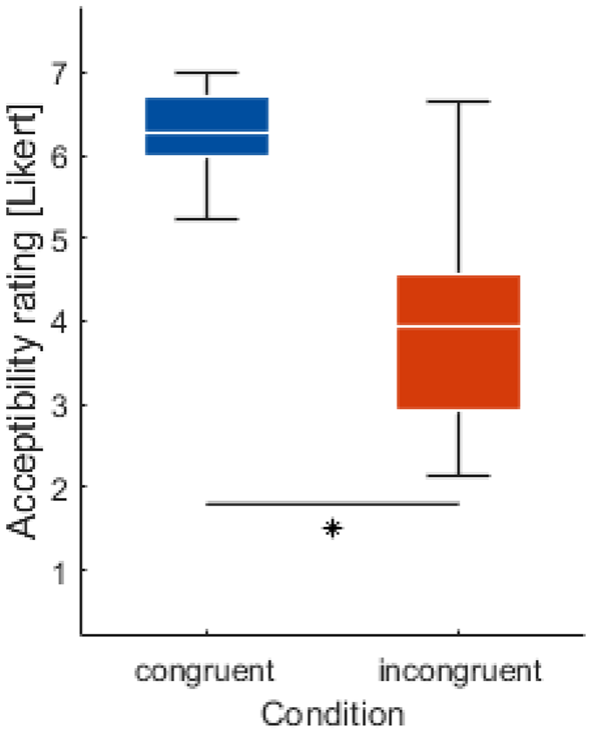
Acceptability ratings after each trial (see “Methods”). The Likert scale ranged from ’completely acceptable’ (7) to ’completely unacceptable’ (1). The *Condition* congruent is always shown in blue and incongruent in red. On each box, the central marks indicate the median, and the bottom and top edges of the boxes indicate the 25th and 75th percentiles, respectively. Statistical significance is indicated with *.

Overall, participants rated the *Acceptability* of the AV’s behavior higher when it was congruent with their own decision ($t(32)=12.02, p<.001, d=2.09$) (see Fig. 1).

Regarding the question after debriefing, if they knew they were not interacting with a real artificial intelligence (AI), 23.5% indicated “yes”, 44.1% “not sure”, and 32.4% “no”.

### EEG results

The task was designed such that out of a total of 200 trials, the AV behaved congruently with the participant’s decision in 50% and incongruently in 50% of the trials. Additionally, in 50% of the trials, the AV turned through the proposed gap in traffic while in the other 50%, it waited for another gap. This resulted in different numbers of trials in each combination in the final analysis: After artifact correction, in $34.86 \% (\bar{x} = 68.73 (SD=23.13))$ the AV congruently turned, $14.92 \% (\bar{x} = 29.58 (SD=24.30))$ the AV incongruently turned, $15.13 \% (\bar{x} = 29.97 (SD=23.56))$ the AV congruently waited, and $35.09 \% (\bar{x} = 69.18 (SD=23.88))$ the AV incongruently waited (see supplementary Table 1). After artifact rejection, there were on average $\bar{x} = 98.70 (SD=1.31)$ congruent trials, and $\bar{x} = 98.76 (SD=1.75)$ incongruent trials (see supplementary Table 1) per participant for the final analysis.

The following section presents the results of the two analysis of variancess (ANOVAs) (*Condition x Electrodes*) conducted to analyze the N1, P2, N2, and P3 components of the ERP. To control the false discovery rate only $p<0.0167$ is considered significant^35^.

#### **N1 results**

**Fig. 2:**
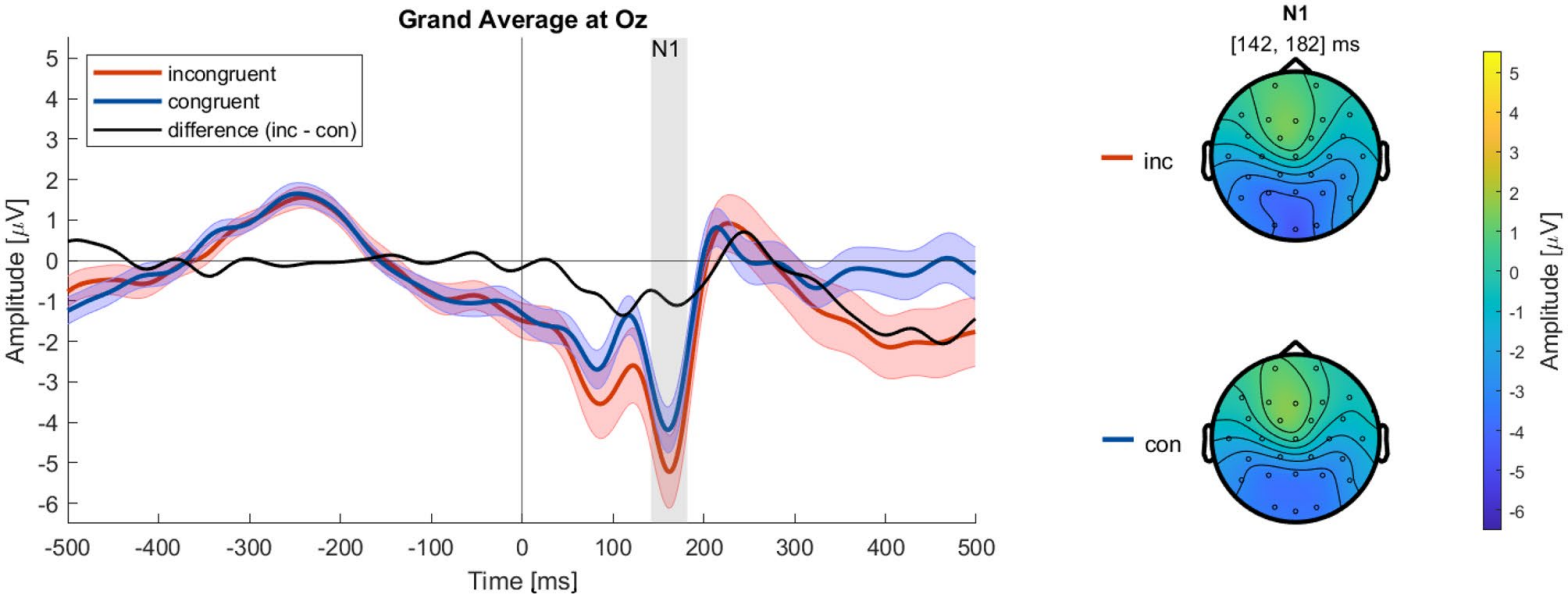
The grand average ERPs at electrode Oz time-locked to the presentation of the AV’s decision (see “Methods”) for both conditions, incongruent (red), congruent (blue), as well as their difference (black), were filtered from 0.1 to 20 Hertz (Hz) and with a baseline from – 500 to 0 ms. The shaded error bars indicate the SE. The gray shading indicates the identified time interval of the N1 component corresponding to the topographies. The topographies show the N1 in the incongruent , and congruent condition, sharing one color bar.

For the N1 (142 to 182 ms), we found no significant main effect of *Condition* on the amplitude ($\mu$V) $(F_{(1,32)}=9.90, p=.27)$ (see supplementary Table 2). The standard errors (SEs) of the incongruent and congruent N1 amplitude overlap (see Fig. 2).

**Fig. 3:**
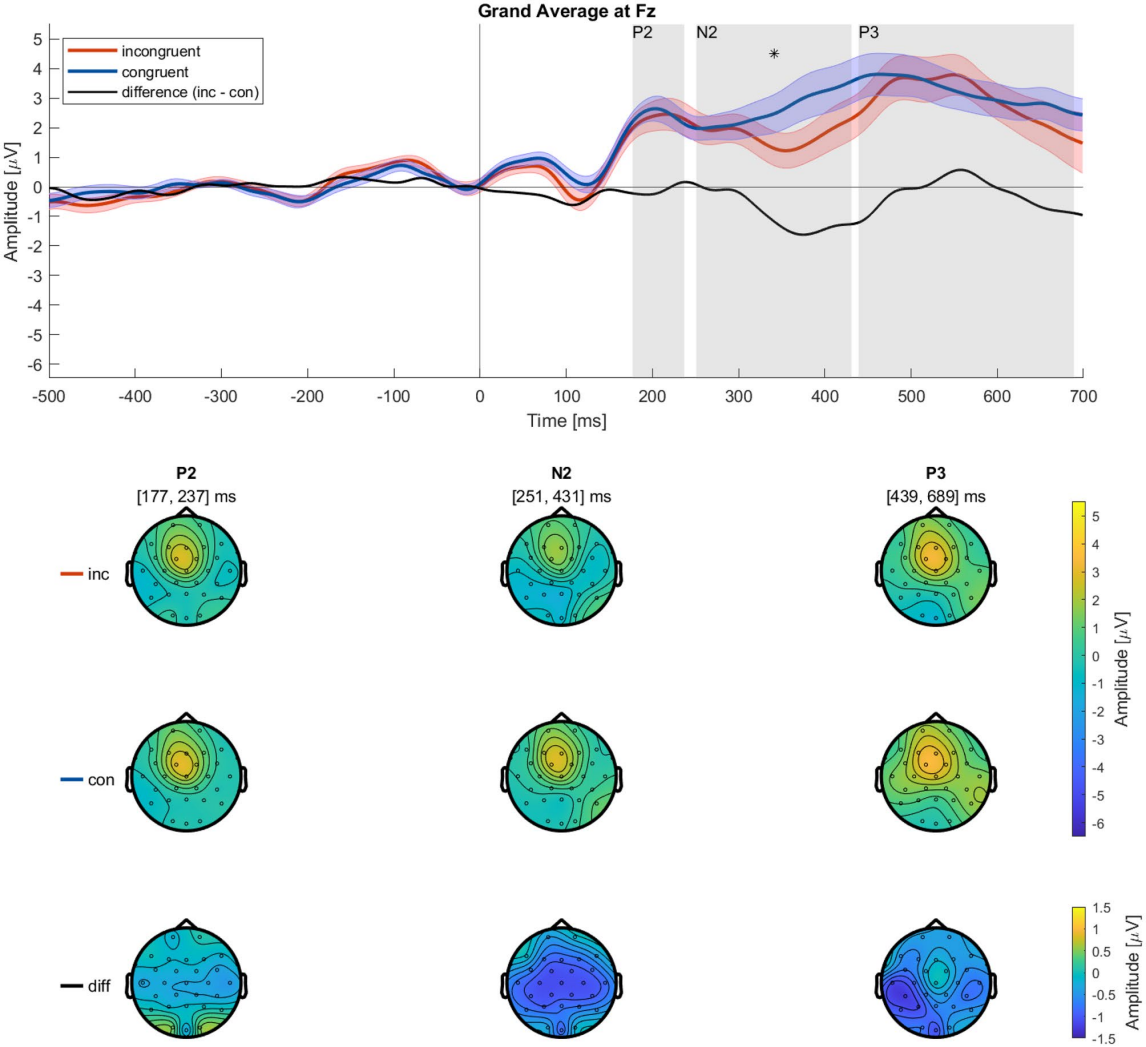
The grand average ERP at electrode Fz time-locked to the presentation of the AV’s decision (see “Methods”) for both conditions, incongruent(red), congruent (blue), as well as their difference (black), were filtered from 0.1 to 20 Hz and with a baseline from − 500 to 0 ms. The shaded error bars indicate the SE. The gray shading indicates the time intervals of the components P2, N2, and P3 corresponding to the topographies below. * indicate significant time intervals. The topographies show the ERP components P2, N2, and P3 column-wise in the given time intervals. The first row shows topographies from the incongruent, and the second row from the congruent condition. The third shows their difference. Note, that the two conditions share one color bar, whereas we used a smaller color bar for the difference between conditions.

There was a significant main effect on *Electrodes* $(F_{(2,96)}=48.24$ $p(Greenhouse-Geisser(GG))<.001$ $\eta _p^2=.60)$ (see supplementary Table 2 & 3). This effect describes the topography over the midline electrodes. As typical for a N1 the amplitude was the lowest at parietal and occipital sites. (see Fig. 3)

Furthermore, there was no two-way interaction *Condition x Electrodes* $(F_{(2,96)}=.92, p_{(GG)}<.43)$ in the N1 time window (see supplementary Table 2).

#### **P2 Results**

For the P2 (177 to 237 ms), we found no significant main effect of *Condition* on the amplitude ($\mu$V) $(F_{(1,32)}=.47, p=.50)$ (see supplementary Table 4). The SEs of the incongruent and congruent P2 amplitude overlap (see Fig. 3).

There was a significant main effect on *Electrodes* $(F_{(2,64)}=26.74, p_{(GG)}<.001, \eta _p^2=.50)$ (see supplementary Table 4 & 5), which again illustrates the topography of this component consisting of an increased amplitude over fronto-central electrodes (see Fig. 3).

Furthermore, there was no two-way interaction *Condition x Electrodes* $(F_{(2,64)}=.50, p_{(GG)}<.52)$ in the P2 time window (see supplementary Table 4).

#### **N2 results**

For the N2 (251 to 431 ms) we found a significant main effect of *Condition* on the amplitude ($\mu$V) $(F_{(1,32)}=15.14, p<.001, \eta _p^2=.32)$ (see supplementary Table 6). The amplitude of the N2 was greater in incongruent vs. congruent trials (see Fig. 3).

There was also a significant main effect on *Electrodes* $(F_{(2,64)}=34.68$ $p(Greenhouse-Geisser(GG))<.001$ $\eta _p^2=.52)$ (see supplementary Table 6 & 7). This effect describes the topography in both conditions, showing the highest amplitude at Fz, and then in descending order at Cz and at Pz.

Furthermore, there was no two-way interaction *Condition x Electrodes* $(F_{(2,64)}=.54, p_{(GG)}<.50)$ in the N2 time window (see supplementary Table 6).

Exploratorily, we conducted a correlation analysis to examine whether a difference in subjective acceptability between congruent and incongruent conditions is significantly associated with a difference in N2 amplitude between those two conditions. Frontally, we find a significant Pearson correlation $(r=.47, p=.006)$, however not centrally $(r = .25, p = .16)$, nor parietally $(r = -.04, p = .83)$.

#### **P3 results**

Similarly to the N1 and P2, for the P3 (439 to 689 ms) we found no significant main effect of *Condition* on the amplitude ($\mu$V) $(F_{(1,32)}=.77, p=.39)$ (see supplementary Table 8). During the P3, the SEs of the incongruent and congruent amplitude overlap (see Fig. 3).

There was a significant main effect on *Electrodes* $(F_{(2,64)}=35.32$ $p(Greenhouse-Geisser(GG))<.001$ $\eta _p^2=.53)$ (see supplementary Table 8 & 9). In the topography, this is visible as the amplitude of the P3 descriptively being higher at Fz and Cz than at Pz (see Fig. 3).

Furthermore, there was no two-way interaction *Condition x Electrodes* $(F_{(2,64)}=3.39, p_{(GG)}<.06)$ in the P3 time window (see supplementary Table 8).

## Discussion

In the present study, we investigated modulations of the N1, P2, N2, and P3 components of the ERPs, when an AV maneuvers left turn situations through oncoming traffic.

Regarding the behavioral results, as we expected, participants rated trials where the AV behaved congruent to their assessment as more acceptable than trials with incongruent behavior (see Fig. 1). Note, that the distribution of acceptability rating in the incongruent condition was quite wide. Possibly since AV never produced an accident, some participants rated also incongruent trails as fairly acceptable. This pattern was also present when dividing trials with respect to whether the AV turned or waited: participants rated turning congruently as more acceptable than turning incongruently, and they rated waiting congruently as more acceptable than waiting incongruently (see supplementary Fig. 1).

In the debriefing, less than one in four participants indicated that they at some point knew that they were not interacting with an AI but a random system. However, these participants could still agree or disagree with its behavior. Therefore, we kept these participants for the analysis to avoid suffering from a lower experimental power.

Overall, participants demonstrated expected behavior, which leads us to infer that participants engaged thoroughly with the task, thus serving as a foundation for the following EEG interpretation.

**Fig. 4:**
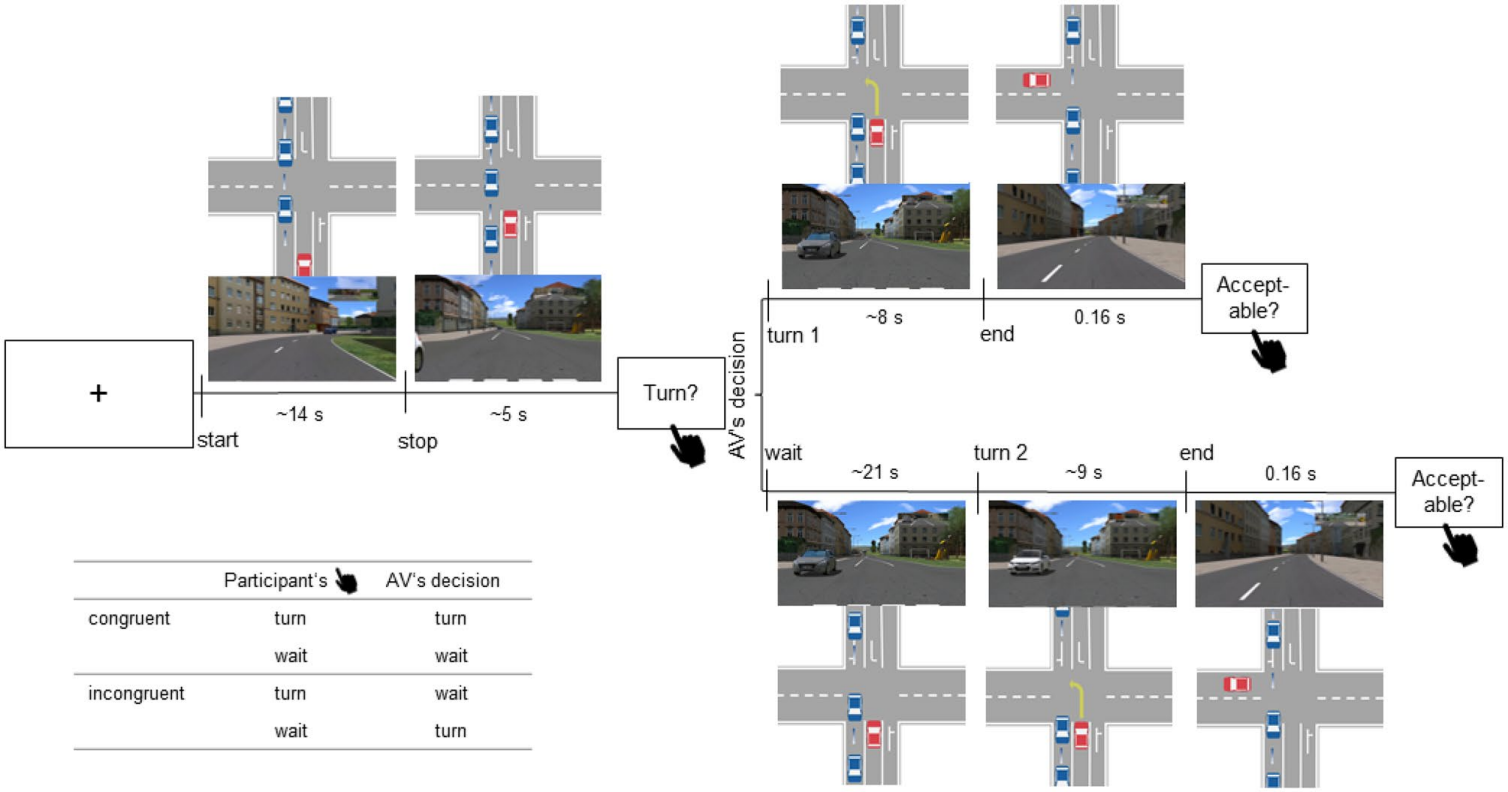
Schematic overview of an exemplary trial in the LT task. The flow chart illustrates the trial’s time course, with all relevant events marked by vertically labeled ticks. For each relevant event, a screenshot from a corresponding video and a schematic drawing of the traffic situation at that moment are provided. The videos show the driver’s perspective of the red vehicle in the schematic drawings. Each trial begins with a cross at the center of the screen, then the AV approaches the crossing (start) and stops (stop). At the first possible gap between oncoming vehicles (blue vehicles in the schematic drawings), participants were asked to indicate via button press whether they wanted to turn left through the gap (Turn?). As illustrated in the table, in congruent trials the AV’s decision complies, in incongruent trials it diverges from the participants’ decision. The AV either turns (turn 1) or waits (wait) for the next possible gap, where it eventually turns (turn 2). The videos ended after a successful turn (end). At the end of each trial, participants were asked to rate the acceptability of the AV’s behavior (Acceptable?). See supplementary Methods for the exact wording of the used items. On each section of the timeline, the mean duration of each phase is indicated in s.

In the ERP time-locked to the AV’s decision (see Fig. 4), there was a significant main effect of *Condition* in the N2 component, but not in N1, P2, and P3. In the ERP components N2 descriptively, there was an increased negativity in the incongruent compared to the congruent *Condition* (see Fig. 3).

In contrast to the majority of ERP studies, we used dynamic instead of static stimulus material, specifically videos generated in a driving simulation environment showing left turn maneuvers by an AV through oncoming traffic. Therefore, we analyzed early components to confirm if we can obtain stable ERPs with our material. Even though there was no difference between conditions in the N1, the topographies (see Fig. 2) reveal the typical pattern of an occipital negativity^36^. We interpret this as a strong indicator of the credibility of our ERP data. The N1 is thought to play a crucial role in visual attention and as an index of discrimination processes^30^. Further, it is associated with the processing of spatial visual information^37^, which is relevant to successfully assessing traffic situations. However, as the N1 is an early ERP component, in this case, it likely reflects differences in the physicality of the stimulus material (size, position, contrast, etc.)^38,39^, and therefore, here needs to be interpreted with caution.

Regarding the subsequent components, P2, N2, and P3, their topographies (see Fig. 3) all showed increased amplitudes in fronto-central sites, whereas the amplitudes in parietal and occipital sites appeared lower. Visibly, these topographies resemble the typical topographies of a P3a^40^ and this dominant topography already builds up during the earlier components. Hence, when further interpreting the N2 we concentrated on the difference topography contrasting incongruent - congruent trials (see Fig. 3) because in the ERP plot (see Fig. 3) the N2 negativity is only prominently visible in the incongruent condition (red line) but not in the congruent (blue line). Therefore, the difference topography incongruent - congruent of the N2 likely reflects the actual N2 topography, isolated from the ramping up towards the P3a. The observed N2 shows a central distribution spreading widely over frontal, parietal, as well as lateralized sites (see Fig. 3). Additioally, we showed a positive correlation between N2 difference and Acceptibility rating difference. Participants who rate incongruent trails as less acceptable display a more negative N2 amplitude. The N2 ERP component was shown to have different generators and functions with distinct topographies depending on specific task modalities^20^. Roughly, the N2 is frequently subdivided into (1) the anterior or fronto-central N2 in response to deviating non-targets, and response inhibition, (2) the posterior N2, in response to deviant targets, and (3) the posterior contra-lateral N2 (N2pc), a marker of spatial visual attention^20,33^. The wide distribution in the present topography, however, suggests that not one isolated function was necessary to solve our task, but rather a combination of anterior and posterior N2 was at play. As the N2 is especially sensitive to incongruent stimuli and task modalities^20,41–47^, and significantly correlated with the Acceptability ratings we would argue that the N2 might be a helpful indicator when identifying critical situations, even in complex stimulus material such as traffic using ERPs.

Contrary to our hypothesis, there were no differences between the *Conditions* regarding the amplitudes of both ERP components P2 and P3. In both conditions, the P2 and P3 peaks are located fronto-centrally (see Fig. 3). The prefrontal P2 is thought to play a role in stimulus-response mapping, in other words, classifying stimuli and matching them to the correct response^31^. The lack of differences in P2 amplitudes in our conditions suggests that this functionality was equally important during congruent as well as during incongruent trials.

For the P3a, we found a fronto-central topography that did not differ between conditions. The P3a is usually associated with orienting attention to significant events, whereas the P3b is associated with updating of working memory^20^. This might suggest that an AV executing a left turn through oncoming traffic by itself represents a significant event.

To address some limitations, in the present study, we focused on plausible traffic situations for an AV with approval for road use. Hence, our simulation did not entail truly failed traffic maneuvers (accidents), since for those it is unambiguously clear that they are unacceptable. Further, accidents are difficult to plausibly simulate, and they should already be minimized by manufacturers, especially since there is evidence showing that for future AV users adoption is tied to substantially increased safety (4–5 times safer) in comparison to human drivers^48^. The simulated AV in this study always turns successfully and never has an accident. Over time, participants more frequently indicated that they wanted to turn even through the smaller gaps between vehicles. This suggests that over system usage time, participants showed increased trust towards the AV, which could be interpreted as a positive sign for future acceptance of AV. Additionally, the fact that the AV always performed a successful turn maneuver resulted in a wide distribution of acceptability ratings in the incongruent condition. Hence, there might be different levels of acceptability in such human-AV interaction situations. Thus, it might be worthwhile to differentiate those in a future experimental design. Further, this study was designed in such a fashion that the AV behaved congruently to the participant’s assessment in 50% of trials and incongruently in 50%. Consequently, in the congruent condition, there was an increased number of trials where the AV turned, whereas, in the incongruent condition, there was an increased number of trials where the AV waited for another gap. Therefore, the observed increase of the N2 response is particularly noteworthy: It occurred when the AV did not show the expected behavior (turning), which would have resulted in immense changes in the visual field, but waited - so participants’ visual perspective remained the same in most incongruent trials. However, the impact of the changes in the visual field on our effect could be a subject for further study. Taken together, the significant event was the AV not showing an action but remaining inactive. We find this a promising indication that in the future successful ERP-based brain computer interface (BCI) applications in the context of AVs.

To give some future outlook, in the present study, we set out to investigate the usefulness of ERPs in the context of identifying critical situations with AVs. For this purpose, we decided to start in a rather controlled laboratory environment. We could show that it is possible to find high-quality ERPs even in complex moving stimulus material. In the next steps, the reliability of our findings would need to be tested in setups closer to field application: e.g., in a Wizard of Oz environment in an actual car and using smaller, mobile EEG devices. Further, to develop an actual ERP based device to identify unacceptable traffic situations, it would be necessary to employ single-trial classifiers (e.g.,^25,49–53^). In all those studies, the classifiers have been fed with training data from situations providing clear wrong and right outcomes. Since traffic is often more ambiguous, it might be advisable to include data from more frequently occurring stimulus material, such as ours, in these lines of research. However, this was beyond the scope of the current study and is a wide field for further research.

In conclusion, we investigated the usefulness of of ERPs in the context of AVs. In our controlled laboratory environment driving simulation study, we could show that it is possible to find high quality ERPs even in moving stimulus material. When observing an AV executing a traffic maneuver in a fashion that contradicts participants’ assessment, we find increased amplitudes in the ERP components N1 and N2, but no significant difference in P2 and P3. This suggests that in human-AV interactions, it might be possible to use ERP based devices to identify critical situations. However, further research is needed to bring current findings from fundamental research closer to application.

## Methods

Note, that this is the same set of participants, procedure, and apparatus than in our previous Moral Machine (MM) study^29^ (under revision), because both studies were recorded simultaneously.

### Participants

Thirty-six volunteers were recruited to participate in this study to achieve sufficient statistical power in our design. Participants were included in the study if they were over 18 years old, right-handed, native German speakers, had normal or corrected to normal vision, and had no psychiatric or neurological diseases. Additionally, participants were required to have a valid German driver’s license and be legally fit to drive under German regulations (§31 StVZO, and §316 StGB) at the time of the experiment. All participants gave written informed consent and received 10 € per hour for participation. The research protocols were approved by the Commission for Research Impact Assessment and Ethics (“Kommission für Forschungsfolgenabschätzung und Ethik”) at the Carl von Ossietzky University of Oldenburg and complied with all relevant ethical regulations. Two participants were excluded because they participated in only one of the two recording sessions. Another participant was excluded because they clicked ’turn’ in all trials. Hence, there were no ’congruent wait’ and ’incongruent turn’ trials for that participant. Therefore, the data of 33 participants (mean age = 24.455, SD = 3.042, age = 3.042, age range = 19–34 years, male = 18) were included in the analysis.

### Procedure

The experiment consisted of two sessions, conducted on different days (days between sessions: mean=6.618, SD=8.707). Each session took $\sim 150-200$ min.

During the first experimental session, participants gave written informed consent and filled in the demographic questionnaires. In both sessions, the EEG and the peripheral physiological sensors were positioned. Participants were then seated in an electrically shielded, dimly lit chamber at $\sim 120$ cm distance in front of a display. First, participants were familiarized with the two tasks, Left Turn (LT), and MM, in a short training. To achieve somewhat similar training times, for the LT task there were three congruent trials, and for the MM task there were six congruent trials.

The main experiment consisted of both tasks. The order of the tasks was counterbalanced both between sessions and participants. The current paper discusses the data from the LT task. For results from the MM task see^29^ (under revision). The LT task of the main experiment was divided into four ($\sim 20$ min) blocks. Between blocks, participants had the opportunity to take short, self-paced breaks. After the second session, they were debriefed that they did not interact with an AI but a deterministic abstract machine. Then they were asked if they knew they were not interacting with a real AI, with the answering options “yes”, “not sure”, and “no”. Table 1 summarizes the overall procedure.

**Table 1:** Overview of procedure.

	Session I				Session II			
Task	LT training	LT	MM training	MM	MM training	MM	LT training	LT
Conditions	con	con & inc	con	con & inc	con	con & inc	con	con & inc
Trials	3	100 (in 4 blocks)	6	100 (in 2 blocks)	6	100 (in 2 blocks)	3	100 (in 4 blocks)
Time [min]	$\sim$3	$\sim$80 (in 4 blocks)	$\sim$1	$\sim$20 (in 2 blocks)	$\sim$1	$\sim$20 (in 2 blocks)	$\sim$3	$\sim$80 (in 4 blocks)

### Left turn task

For both tasks, there was a cover story that the AV would make elaborate, data-driven decisions. In the debriefing at the end of the last experimental session, it was clarified that all of the AV’s supposed decisions were predetermined by a random number generator.

Each trial began with a cross that was presented at the center of the screen for 1000 ms. Then, participants saw short video sequences from the driver’s perspective of an AV approaching an intersection with oncoming traffic. At the stop line, the simulation was interrupted and participants were asked to assess whether they would turn left in the next gap between oncoming vehicles or whether they would prefer to wait. Participants were instructed to ’answer from their gut’ when they were ready and to indicate their opinion using the left and right arrow buttons on a standard keyboard. After pressing an arrow, participants viewed a video in which the AV behaved either congruently or incongruently with their decision. After each trial, participants rated the acceptability of the AV ’s decision on a Likert scale ranging from 1, completely unacceptable, to 7, completely acceptable. This range was chosen to include a neutral middle point (4) while ensuring that differences remain distinguishable in the human working memory^54^. See Fig. 4 for an overview of the task.

We created 30 different vignettes. Each participant performed 200 trials in total in random order. Half of the trials were presented in the congruent setting, and half in the incongruent setting. Thus, each scenario was presented at least 3x in a congruent and 3x in an incongruent setting (see Table 1).

### Apparatus

#### **Hardware**

The stimulus material was presented on a Samsung SyncMaster display: P247GH, 1920 x 1080 pixels, 60 Hz refresh rate.

The EEG data were recorded using a 32-channel actiCAP snap electrode system with the standard 10-10 layout (BrainProducts GmbH, Gilching, Germany). The reference electrode was placed on the tip of the nose and a vertical electro ecculogramm (EOG) electrode was placed under the right eye. The ground electrode was placed at electrode location FPz. Thus, the final EEG signal was acquired from 30 active Ag-AgCl electrodes. Impedance was measured before recording and kept below 20 k$\Omega$. EEG signals were digitized at a rate of 1000 Hz by a 16-bit ActiChamp EEG amplifier.

#### **Software**

The task was programmed using the Psychtoolbox^55–57^ in MATLAB 2021a (The MathWorks, Inc., Natick, MA, USA).

The video sequences (see Fig. 4 for screenshots) were created in the commercial driving simulation software SILAB Version 6 (WIVW GmbH, Würzburg, Germany).

Physiological signals were recorded using BrainVision Pycorder software (BrainProducts GmbH, Gilching, Germany).

Data preprocessing and plotting were performed using the FieldTrip toolbox (^58^; http://fieldtriptoolbox.org) in MATLAB 2022b (The MathWorks, Inc., Natick, MA, USA).

All statistical analyses were computed using SPSS 29 (IBM, Armonk, NY, USA).

### Data analysis

#### **EEG preprocessing**

The EEG data were then demeaned, detrended, and filtered between 1 and 35 Hz using a Butterworth filter (ft_preprocessing)^58^. The data were then segmented into 1000 ms epochs without overlap. Epochs containing data that exceeded a threshold of 1000 $\mu$V were rejected. Then, an independent component analysis (ICA) was performed, and components containing eye and muscle movement artifacts were visually identified and rejected. The remaining components were projected back onto the raw EEG data.

The raw data were then filtered from 0.1 to 35 Hz, and cut around the trigger at “AV’s decision” from − 2500 to 2500 ms (see Fig. 4). This trigger marked the first frame in the video where the motion of the vehicle differed in either the car remaining still or starting to move. Using accumulated z-score thresholding (ft_artifact_zvalue)^58^, trials with a $z \ge \ 20$ were rejected. The trials from the two experimental sessions were merged, and the data were downsampled to 250 Hz. Since our stimulus material was dynamic throughout, which might have caused the baseline to be unstable, we accounted for this by applying a wide baseline correction window from -500 to 0 ms. For the ERPs, we averaged the epochs from each *AV’s decision* (see Fig. 4) for each participant. Then we calculated a grand average over participants for each condition separately (congruent, and incongruent).

To identify the ’N1’, ’P2’, ’N2’, and ’P3’ components of the ERP, we applied a peak picking algorithm (findpeaks()) on the grand average collapsed over all conditions. For each component, we defined a time window and target electrodes for identifying the peak or trough amplitudes ($\mu$V) respectively: N1 (100-200 ms at Oz)^33^, P2 (150-250 ms at Fz)^59^, N2 (200-350 ms at Fz)^20^, and P3 (250-500 ms at Cz)^40^. For each component, we defined a relevant time window around the identified peaks or troughs: N1 ($162\pm 20$ ms), P2 ($207\pm 30$ ms), N2 ($341\pm 90$ ms), and P3 ($489-50+200$ ms). Note, that the windows’ widths were chosen to ensure that they covered the whole component. Subsequently, the P3 time window was shifted to the right to ensure that there was no overlap with the N2 window and to better capture the skewed shape of the P3 (see gray shadings in Fig. 3). These four component windows were then applied to the previously calculated grand averages for the conditions (congruent and incongruent) All samples in one time window were collapsed for the three relevant electrodes (Fz, Cz, Pz) and exported for inference statistical analyses.

#### **Statistical analysis**

To examine the differences in acceptability ratings between conditions, we conducted a repeated measures t-test.

To examine the differences in the ERP components between conditions, we conducted four two-way repeated measures ANOVAs with the factors *Condition* (congruent, incongruent) x *Electrodes* (Fz, Cz, Pz), one for each relevant component N1, P2, N2, and P3. Note that for N1, we also included Oz in the analysis. To account for the sphericity problem GG corrections were used to correct the df. Within each of these four ANOVAs, we controlled the false discovery rate^35^. Accordingly, we applied adjusted $\alpha$ levels to the three ANOVA effects to determine significance: .0500 for the largest p value, .0333 for the middle p value, and .0167 for the smallest p value^35^.

Each ANOVA archives a strong statistical power of $1-\beta \ge .94$ with 33 complete datasets, and a conservatively assumed correlation between repeated measures of $r =.2$, and a conventionally medium-sized effect $(f =.25)$^60–62^ (see supplementary Methods for details).

Furthermore, as a post-hoc procedure to further disentangle significant three-level main effects and interaction effects, we employed Bonferroni corrected t-tests for *C* relevant comparisons on the simple main effects.

Exploratorily, we performed a Pearson correlation analysis between N2 amplitude differences ($N2_{incongruent} - N2_{congruent}$) and difference acceptability ratings ($incongruent - congruent$).

## Supplementary Information

Supplementary Information.

## Data Availability

The data are available from the corresponding author upon reasonable request.
