# Supplementary material for: Detecting unacceptable behavior of an autonomous vehicle using electroencephalography
Source: Sci Rep. 2025 Sep 12;15:32462. doi: 10.1038/s41598-025-18305-2 (PMC12432218; doi:10.1038/s41598-025-18305-2)
Supplement: Supplementary file 1 — Supplementary Information. [file 41598_2025_18305_MOESM1_ESM.pdf]

Supplementary Material to "Detecting Unacceptable Behavior of an Autonomous Vehicle Using Electroencephalography"

Maren A. K. Bertheau[1,2] and Christoph S. Herrmann[1,3,4,*]

[1]Department of Psychology, Experimental Psychology Lab, Carl-von-Ossietzky University, Ammerländer Heerstr. 114-118, 26111 Oldenburg, Germany
[2]Department of Informatics, Karlsruhe Institute of Technology (KIT), Am Fasanengarten 5, 76131 Karlsruhe, Germany
[3]European Medical School, Cluster for Excellence “Hearing for All”, Research Center Neurosensory Science, Carl-von-Ossietzky University, Carl-von-Ossietzky-Straße 9-11, 26129 Oldenburg, Germany
[4]Research Center Neurosensory Science, Carl von Ossietzky University, 26129 Oldenburg, Germany
[*]christoph.herrmann@uni-oldenburg.de

ABSTRACT

This supplementary material contains further details on Results and Methods for the study "Detecting Unacceptable Behavior of an autonomous vehicle (AV) using electroencephalography (EEG)".
Regarding Results, we supplement a table on the number of trials for all condition combinations after artifact correction (see Table 1), the behavioral results from the Likert acceptability item detailed for 'turn' and 'wait' (see Fig. 1), the four two-way analysis of variances (ANOVA) for the components 'N1', 'P2', 'N2', and 'P3' as well as the corresponding post hoc procedures for those effects which showed to be significant in the ANOVA (see Tables 2-9).
Regarding Methods we give further details on technical equipment, the used question items, and the statistical power estimation (see Table **??**).

Results

	congruent			incongruent			total		
	$\bar{x}$	(standard deviation (SD))	%	$\bar{x}$	(SD)	%	$\bar{x}$	(SD)	%
turn	68.7	(23.1)	34.9	29.6	(24.3)	14.9	98.3	(3.7)	49.8
wait	30.0	(23.6)	15.1	69.2	(23.9)	35.1	99.2	(3.4)	50.2
total	98.7	1.3	50.0	98.8	1.8	50.0	197.5	2.8	100

Supplementary Table 1 Average number of trials per participant ($\bar{x}$) for all condition combinations after artifact correction

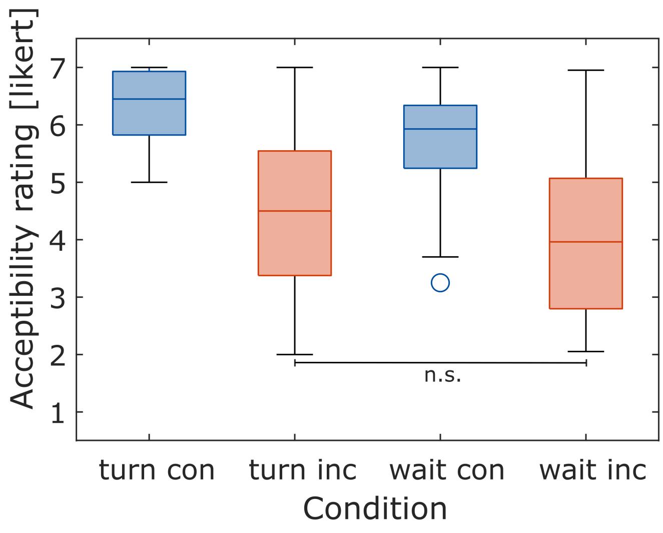

Supplementary Fig. 1 Acceptability ratings after each trial split up for the conditions congruent (blue), and incongruent (red) as well as the AV's action (turn vs. wait). The Likert scale was coded as 'completely acceptable' (7) to 'completely unacceptable' (1). On each box, the central mark indicates the median, and the bottom and top edges of the box indicate the 25th and 75th percentiles, respectively. The only not significant difference is marked (n.s.). All other differences are significant.

Factor	sphericity correction	square sum	df	MSS	F	p	partial η^2
Condition	-	9.895	1	9.895	1.272	0.268	0.038
error(Condition)	-	248.909	32	7.778			
Electrodes	-	1443.616	2	481.205	48.238	<0.001	0.601
	Greenhouse-Geisser (GG)	1443.616	1.929	748.400	48.238	<0.001*	0.601
error(Electrodes)	-	957.664	96	9.976			
	GG	957.664	61.726	15.515			
Condition * Electrodes	-	7.645	3	2.548	0.922	0.433	0.028
	GG	7.645	1.198	6.383	0.922	0.360	0.028
error(Condition*Electrodes)	-	265.347	96	2.764			
	GG	265.347	38.328	6.923			

Supplementary Table 2 Two-way ANOVA for N1 component with GG correction

simple main effect		diff. between means	standard error (SE)	95% confidence interval for difference lower bound	upper bound	significance p	p*C=6
Fz-	Cz	1.169	0.340	0.476	1.862	0.002	0.010*
Fz-	Pz	4.792	0.557	3.657	5.927	<0.001	<0.001*
Fz-	Oz	5.525	0.679	4.141	6.909	<0.001	<0.001*
Cz-	Pz	3.622	0.371	2.868	4.377	<0.001	<0.001*
Cz-	Oz	4,356	0.692	2.946	5.766	<0.001	<0.001*
Pz-	Oz	0.734	0.556	-0.399	1.866	0.196	1

Supplementary Table 3 Bonferroni corrected t-tests with C= 3 relevant comparisons as post hoc procedure for the three level main effect Electrodes at the component N1

Factor	sphericity correction	square sum	df	MSS	F	p	partial η^2
Condition	-	1.815	1	1.815	0.467	0.499	0.014
error(Condition)	-	124.272	32	3.883			
Electrodes	-	303.085	2	151.543	26.740	<0.001	0.455
	GG	303.085	1.518	199.700	26.740	<0.001*	0.455
error(Electrodes)	-	362.711	64	5.667			
	GG	362.711	48.567	7.468			
Condition * Electrodes	-	0.402	2	0.201	0.504	0.607	0.015
	GG	0.402	1.262	0.318	0.504	0.524	0.015
error(Condition*Electrodes)	-	25.530	64	0.399			
	GG	25.530	40.381	0.632			

Supplementary Table 4 Two-way ANOVA for P2 component with GG correction

simple main effect		diff. between means	SE	95% confidence interval for difference lower bound	upper bound	significance p	p*C=3
Fz-	Cz	0.147	0.322	-0.510	0.804	0.651	1.954
Fz-	Pz	2.695	0.515	1.645	3.745	<0.001	<0.001*
Cz-	Pz	2.548	0.382	1.770	3.326	0.002	0.005*

Supplementary Table 5 Bonferroni corrected t-tests with C= 3 relevant comparisons as post hoc procedure for the three level main effect Electrodes at the component P2

Factor	sphericity correction	square sum	df	MSS	F	p	partial η^2
Condition	-	42.908	1	42.908	15.135	<0.001*	0.321
error(Condition)	-	90.720	32	2.835			
Electrodes	-	340.649	2	170.325	34.677	<0.001	0.520
	GG	340.649	1.596	213.390	34.677	<0.001*	0.520
error(Electrodes)	-	314.350	64	4.912			
	GG	314.350	51.084	6.154			
Condition * Electrodes	-	0.665	2	0.332	0.536	0.588	0.016
	GG	0.665	1.200	0.554	0.536	0.500	0.016
error(Condition*Electrodes)	-	39.710	64	0.620			
	GG	39.710	38.402	1.034			

Supplementary Table 6 Two-way ANOVA for N2 component with GG correction

simple main effect		diff. between means	SE	95% confidence interval for difference lower bound	upper bound	significance p	p*C=3
Fz-	Cz	0.768	0.307	0.141	1.394	0.018	0.054
Fz-	Pz	3.086	0.470	2.129	4.042	<0.001	<0.001*
Cz-	Pz	2.318	0.362	1.580	3.056	<0.001	<0.001*

Supplementary Table 7 Bonferroni corrected t-tests with C= 3 relevant comparisons as post hoc procedure for the three level main effect Electrodes at the component N2

Factor	sphericity correction	square sum	df	MSS	F	p	partial η^2
Condition	-	4.502	1	4.502	0.767	0.388	0.023
error(Condition)	-	187.857	32	5.871			
Electrodes	-	329.299	2	164.650	35.318	<0.001	0.525
	GG	329.299	1.541	213.667	35.318	<0.001*	0.525
error(Electrodes)	-	298.364	64	4.662			
	GG	298.364	49.318	6.050			
Condition * Electrodes	-	2.969	2	1.484	3.388	0.040	0.096
	GG	2.969	1.410	2.105	3.388	0.058	0.096
error(Condition*Electrodes)	-	28.039	64	0.438			
	GG	28.039	45.122	0.621			

Supplementary Table 8 Two-way ANOVA for P3 component with GG correction

simple main effect		diff. between means	SE	95% confidence interval for difference		significance	
				lower bound	upper bound	p	p*C=3
Fz-	Cz	0.431	0.316	-0.213	1.076	0.183	0.548
Fz-	Pz	2.926	0.467	1.974	3.877	<0.001	<0.001*
Cz-	Pz	2.495	0.325	1.833	3.156	<0.001	<0.001*

Supplementary Table 9 Bonferroni corrected t-tests with C= 3 relevant comparisons as post hoc procedure for the three level main effect Electrodes at the component P3

Methods

Exact wording of the used items

Turn?

German: 'Möchten Sie in die kommende Lücke abbiegen (linke Pfeiltatste) oder warten (rechte Pfeiltatste)?'
English: 'Do you want to turn into the upcoming gap? turn (left arrow) or wait (right arrow)'

Acceptable?

German: 'Wie akzeptabel fanden Sie das Verhalten des selbst-fahrenden Fahrzeugs?
völlig unakzeptabel 1 - 2 - 3 - 4 - 5 - 6 - 7 völlig akzeptabel' English: 'How acceptable did you find the behavior of the self-driving vehicle?
completely unacceptable 1 - 2 - 3 - 4 - 5 - 6 - 7 completely acceptable'

Statistical power

G*power Version 3 [1, 2] does not allow for power estimations of ANOVAs with repeated measures on two factors [3]. To evade this we adjusted the effect sizes f according to the method suggested by Rasch, Friese, Hofmann & Naumann [3] (Chapter 3.3). For two-factor ANOVAs, they suggest adjusting f based on the other factor: $f' = \sqrt{q} * f$ or $f' = \sqrt{p} * f$, respectively. Statistical power $(1-\beta)$ estimations were carried out for all three effects of the 2x3 ANOVA *Condition x Electrodes* with 33 complete datasets, and a conservatively assumed correlation between repeated measures of $r = .2$. We employed the effect conventions suggested by G*Power: small $(f = 0.10)$, medium $(f = 0.25)$, and large $(f = 0.40)$. For the main effect *Condition* (congruent, incongruent: p = 2) we employed the following adjustment $f' = \sqrt{q} * f$ to G*Power and found:

- small: $f' = \sqrt{3} * 0.1 = 0.173, \lambda = 2.475, 1-\beta = 0.332$
- medium: $f' = \sqrt{3} * 0.25 = 0.433, \lambda = 15.468, 1-\beta = 0.968$
- large: $f' = \sqrt{3} * 0.4 = 0.693, \lambda = 39.598, 1-\beta = 0.999$

For the main effect *Electrodes* (frontal, central, parietal: q = 3) and the interaction *Condition x Electrodes* we employed the following adjustment $f' = \sqrt{p} * f$ to G*Power and found:

- small: $f' = \sqrt{2} * 0.1 = 0.141, \lambda = 2.474, 1-\beta = 0.260$
- medium: $f' = \sqrt{2} * 0.25 = 0.354, \lambda = 15.473, 1-\beta = 0.940$
- large: $f' = \sqrt{2} * 0.4 = 0.566, \lambda = 39.602, 1-\beta = 0.999$

Thus, overall we at least achieved statistical power of $1-\beta = 0.940$ for the three ANOVA effects assuming a medium sized effect.

References

1. Faul, F., Erdfelder, E., Lang, A.-G. & Buchner, A. G*Power 3: A flexible statistical power analysis program for the social, behavioral, and biomedical sciences. *Behavior Research Methods* **39,** 175–191 (2007).
2. Faul, F., Erdfelder, E., Buchner, A. & Lang, A.-G. Statistical power analyses using G*Power 3.1: Tests for correlation and regression analyses. *Behavior Research Methods* **41,** 1149–1160 (2009).
3. Rasch, B., Friese, M., Hofmann, W. & Naumann, E. *Quantitative Methoden. Band 2* 5th ed. Supplementary material retrieved from `https://www.lehrbuch-psychologie.springernature.com/sites/default/files/2022-01/Rasch_A5_978-3-662-63283-3_Kapitel_3_GPower_R_Ergaenzung.pdf` (Springer, Heidelberg, 2021).
